# Identification of repressive and active epigenetic marks and nuclear bodies in *Entamoeba histolytica*

**DOI:** 10.1186/s13071-016-1298-7

**Published:** 2016-01-14

**Authors:** Daniela Lozano-Amado, Abril Marcela Herrera-Solorio, Jesús Valdés, Leticia Alemán-Lazarini, Ma. de Jesús Almaraz-Barrera, Eva Luna-Rivera, Miguel Vargas, Rosaura Hernández-Rivas

**Affiliations:** Molecular Biomedicine Department, Centro de Investigación y de Estudios Avanzados del Instituto Politécnico Nacional (IPN), Av. Instituto Politécnico Nacional # 2508, Apartado postal 14–740,, 07360 D. F. Mexico, México; Biochemistry Department, Centro de Investigación y de Estudios Avanzados del Instituto Politécnico Nacional (IPN), Av. Instituto Politécnico Nacional # 2508, Apartado postal 14–740,, 07360 D. F. Mexico, México

**Keywords:** *Entamoeba histolytica*, Transcriptional regulation, Histone post-translational modifications, Epigenetics, Nuclear architecture

## Abstract

**Background:**

In human hosts, *Entamoeba histolytica* cysts can develop into trophozoites, suggesting that the life cycle of this parasite are regulated by changes in gene expression. To date, some evidence has suggested that epigenetic mechanisms such as DNA methylation and histone modification are involved in the regulation of gene expression in *Entamoeba*. Some post–translational modifications (PTMs) at the N-terminus of *E. histolytica’s* histones have been reported experimentally, including tri-methylation in the lysine 4 of histone H3 (H3K4me3) and dimethylation in the lysine 27 of histone H3 (H3K27me2), dimethylation of arginine 3 (H4R3me2) and the indirect acetylation of histone H4 in the N-terminal region. However, it is not known which residues of histone H4 are subject to acetylation and/or methylation or where in the nucleus these epigenetic marks are located.

**Methods:**

Histones from trophozoites of *E. histolytica* were obtained and analyzed by LC-MS/MS. WB assays were performed using antibodies against epigenetic marks (acetylated lysines and methylated arginines). Immunofluorescence assays (IFA) were carried out to determine the distribution of PTMs and the localization of DNA methylation as a heterochromatin marker. Nuclear bodies such as the nucleolus were identified by using antibodies against fibrillarin and nucleolin and speckles by using anti-PRP6 antibody.

**Results:**

Some new PTMs in histone H4 of *E. histolytica*, such as the acetylation of lysines 5, 8, 12 and 16 and the monomethylation of arginine 3, were identified by WB. IFA demonstrated that some marks are associated with transcriptional activity (such as acetylation and/or methylation) and that these marks are distributed throughout the *E. histolytica* nucleus. Staining with antibodies against anti-pan-acetylated lysine H4 histone and 5-methyl cytosine showed that the activation and transcriptional repression marks converge. Additionally, two nuclear bodies, the nucleolus and speckles, were identified in this parasite*.*

**Conclusions:**

This study provides the first evidence that the nucleus of *E. histolytica* is not compartmentalized and contains two nuclear bodies, the nucleolus and speckles, the latter of which was not identified previously. The challenge is now to understand how these epigenetic marks and nuclear bodies work together to regulate gene expression in *E. histolytica*.

**Electronic supplementary material:**

The online version of this article (doi:10.1186/s13071-016-1298-7) contains supplementary material, which is available to authorized users.

## Background

The parasite *Entamoeba histolytica* has two morphologically distinct life stages: the cyst, which is the infectious form that transmits disease from person to person, and the trophozoite, which is the invasive form that multiplies in the colon and can eventually invade the liver, brain and lungs. A total of 500 million people worldwide are affected by this parasite; resulting in 50 million cases of invasive disease and approx. 70,000 deaths annually [1].

Despite the medical relevance of *E. histolytica*, very little is known about how gene expression is modulated in this parasite during the invasion of its human host or the encystation process. Changes in the abundance of transcripts in *E. histolytica* are associated with human host invasion [2] and with conversion between the cyst and the trophozoite form. However, the molecular mechanisms that regulate gene expression in this parasite are poorly understood. A number of *cis* elements that function as gene promoters in this parasite and transcription factors that recognize these elements have been described [3]. Additionally, it has been shown that the *E. histolytica* genome is organized into chromatin, whose fundamental unit is the nucleosome [4], and contains genes encoding histones H2A, H2B, H3 and H4. Thus, it is very likely that these histones form the nucleosomes of this parasite. However, the DNA that separates each nucleosome (the DNA linker) exhibits an irregular length compared with the 40 bp DNA linker found in metazoans [4]. Furthermore, it has been found that although the amino - terminus of the *E. histolytica* histones diverge from the primary sequence present in the metazoan histones, they are highly basic and contain several lysine and arginine residues that may be potential targets for post-translational modifications such as acetylation and methylation, through the action of histone acetyltransferases (HATs) or lysine or arginine methyl transferases (HKMTs or PRMTs), respectively [5]. *In silico* analysis of the *E. histolytica* genome has revealed the presence of HAT enzymes belonging to the GNAT and MYST families as well as the presence of a protein capable of removing acetyl groups present at the amino-terminus of histones, a class I histone deacetylase (HDAC) [6]. To date, the only post-translational modifications that have experimentally been shown to occur at the amino-terminus of histone H3 are the di- and tri-methylation of lysine 4 (H3K4me2/3) in *Entamoeba* [7], which are associated with changes in transcriptional activity, as well as the di-methylation of lysine 27, which is highly enriched in genes silenced through RNA interference (RNAi) [8]. In the case of *E. histolytica*, histone H4 shares 71 % identity with the mammalian histone H4 [5]. The differences primarily occur in the amino-terminus of histone H4, where three insertions that do not exist in other eukaryotic histone H4 genes are found. One of these insertions is located at the beginning of the NH_2_-terminus, while the second is located between amino acids 10 and 11, and the third is located after amino acid 14 [5]. This last insertion site merits particular attention due to the presence of three extra lysine residues, which could serve as targets for post-translational modifications such as acetylation or methylation. However, it is not known which residues of histone H4 are subject to acetylation and/or methylation, where in the nucleus these epigenetic marks are located, and what roles they play in the nuclear architecture. To address these questions, western blot (WB) analyses and immunofluorescence assays (IFAs) were performed using commercial antibodies against histone H4. Our data suggest that histone H4 is acetylated at lysine residues (K) K5, K8, K12 and K16 and that arginine 3 is mono-methylated. However, antibodies that recognize tri-methylated K20 did not detect this epigenetic mark at histone H4. Furthermore, IFAs performed with antibodies directed against pan-acetyl histone H4 and monomethyl arginine 3 of histone H4 showed that these epigenetic marks associated with transcriptional activation are distributed throughout the nucleus. A similar distribution pattern was found using an antibody that detects DNA methylation (5-methyl cytosine). Taken together, these data indicate that unlike what has been reported in eukaryotes and other parasites, the nucleus of this parasite is not compartmentalized and contains two types of nuclear bodies: the nucleolus and speckles.

## Methods

### Cell cultures of *E. histolytica*

Trophozoites of *E. histolytica* strain HM1:IMSS were axenically cultured at 37 °C in TYI-S-33 medium and harvested from confluent cultures as described [9].

### Nuclear acid protein extracts

Nuclear proteins were obtained as previously described by Byers et al., 2005 [10] with some modifications. Briefly, 8 × 10^7^ log phase cells were chilled and centrifuged for 5 min at 500 × *g* and washed twice with ice-cold PBS. Trophozoites were resuspended in 1 ml of lysis buffer (10 mM Tris pH 6.5, 27 mM Na_2_S_2_O_5_, 1 % Triton X-100, 10 mM MgCl_2_, 25 mM sucrose) and incubating at 4 °C for 10 min. Trophozoites were lysed by 25 strokes on a prechilled Douncer homogenizer and the sample was centrifuged at 1000 × *g* for 10 min at 4 °C. The supernatant containing the cytoplasmic fraction was recovered and kept at −80 °C. The nuclei were purified by sucrose gradient centrifugation. For this purpose, the nuclei were resuspended in 333 μL ml of extraction buffer (10 mM HEPES pH 7.9, 10 mM KCl, 0.1 mM EDTA pH 8.0, 0.1 mM EGTA pH 8.0) and the resulting suspension was layered on 1 ml of extraction buffer containing 0.34 M sucrose. The sample was centrifuged at 9600 × g for 5 min at 4 °C. The supernatant was discarded, whereas the pellet, corresponding to the nuclear fraction, was resuspended in 100 μl of Tris-EDTA buffer (28.5 mM Tris pH 7.4, 37 mM EDTA) with 0.4 M of HCl and incubating overnight at 4 °C. The acid extract was centrifuged for 10 min at 21,000 × g. 800 μL of ice-cold acetone was added to the supernatant, and the mixture was incubated overnight at −20 °C. Precipitated proteins were collected by centrifugation at 21,000 × g for 15 min, washed once with acetone and air dried. The pellet was resuspended in Tris–HCl pH 8.8 and stored at −20 °C. All buffers used in this protocol contained protease inhibitors (Complete, EDTA-free, Roche).

### Nuclear proteins preparation

8 × 10^7^ log phase cells were lysed as described above, and the nuclear fractions were purified by sucrose gradient centrifugation. The pellet was resuspended in 100 μl of RIPA buffer (50 mM Tris pH 7.4, 150 mM NaCl, 5 mM EDTA, 1 % NP-40, 0.5 % sodium deoxycholate, 0.1 % SDS) keeping it on ice for 30 min, mixing occasionally. The sample was centrifuged at 21,000 × g for 20 min at 4 °C and the supernatant was recovered and stored at −20 °C. All buffers used in this protocol contained protease inhibitors (Complete, EDTA-free, Roche).

### Mass spectrometry analysis of *E. histolytica* histones

Proteins were proteolytically digested in-gel after reduction in 10 mM DTT and alkylation in 55 mM iodoacetamide (both buffered in 50 mM ammonium bicarbonate). Sequencing grade trypsin (250 ng, Promega) in 50 mM ammonium bicarbonate was used to digest protein overnight at 37 °C. Digested peptides were analyzed by LC-MS/MS on a Thermo Scientific Exactive Plus Orbitrap Mass Spectrometer in conjunction with an EASY-nLC II nano UHPLC and Proxeon nanospray source. The digested peptides were loaded on a 100 micron × 25 mm Magic C18 100 Å 5U reverse phase trap where they were desalted online before being separated using a 75 micron × 150 mm Magic C18 200 Å 3U reverse phase column. Peptides were eluted with an increasing percentage of acetonitrile over the course of a 60 min gradient with a flow rate of 300 nl/min. An MS survey scan was obtained for the m/z range 300–1600 and acquired with a resolution of 70,000 and a target of 1 × 10^6^ ions or a maximum injection time of 30 msec. MS/MS spectra were acquired using a top 15 method where the top 15 ions in the MS spectra were subjected to HCD (High Energy Collisional Dissociation). MS/MS spectra were acquired with a resolution of 17,500 and a target of 5 × 10^4 or a maximum injection time of 50 msec. An isolation mass window of 1.6 m/z was used for precursor ion selection, charge states 2–4 were accepted, and a normalized collision energy of 27 % was used for fragmentation. A 5 s duration was used for dynamic exclusion.

Tandem mass spectra were extracted and charge state deconvoluted with Proteome Discoverer (Thermo Scientific) and searched using X! Tandem (The GPM, thegpm.org; version Sledgehammer 2013.09.01.2)). X! Tandem was set to search all proteins in the Uniprot.org *E. histolytica* database (April 21 2015) plus the cRAP database of common laboratory contaminants (www.thegpm.org/crap/; 114 entries), and an equal number of reverse protein sequences (16,138 entries total). X! Tandem was searched with a parent ion mass tolerance of 20 PPM, a fragment ion tolerance of 20 PPM, and trypsin as the digestion enzyme with 1 maximum missed cleavage. Carbamidomethylation of cysteine was specified as a fixed modification. Deamidation of asparagine and glutamine, oxidation of methionine and tryptophan, and Glu- > pyro-Glu, Gln- > pyro-Glu, and ammonia loss of the n-terminus were specified as variable modifications.

Scaffold (version 4.4.0, Proteome Software Inc., Portland, OR) was used to validate MS/MS based protein and peptide identifications. Peptide identifications were accepted if they could be established at greater than 95.0 % probability by the Scaffold Local FDR algorithm. Protein identifications were accepted if they could be established at greater than 79.0 % probability to achieve an FDR less than 2.0 % and contained at least 1 identified peptide. Actual protein and peptides FDRs were 0 %. Protein probabilities were assigned by the Protein Prophet algorithm (Nesvizhskii, Al et al., 2003) [11]. Proteins that contained similar peptides and could not be differentiated based on MS/MS analysis alone were grouped to satisfy the principles of parsimony. Proteins sharing significant peptide evidence were grouped into clusters.

### Immunofluorescence assays

Trophozoites in a logarithmic growth phase were harvested and transferred on glass coverslips coating with poly L - lysine and incubated for 3 h at 37 °C to let them attach to the glass surface. An indirect immunofluorescence assay was performed as follows. Amoebas were fixed and permeabilized with cold methanol-acetone 50:50 for 10 min at room temperature and washed twice with PBS buffer. After, trophozoites were incubated for 1 h with 1 % bovine serum albumin in PBS buffer and samples were reacted with anti-acetyl-histone H4 (Millipore 06-866) 1:1500, anti-histone H4 acetyl K12 antibody (Abcam ab61238) 1:300, anti-histone H4 mono methyl R3 antibody (Abcam ab17339) 1:200, anti-5-methylcytosine antibody (Abcam ab10805) 1:25, anti-lamin B1 antibody (Abcam ab16048) 1:200, anti C23 (H-250) antibody (Santa Cruz sc-13057) 1:100, anti-fibrillarin 1:50 and anti-PRP6 1:50 overnight at 4 °C. Then washed with PBS and incubated for 1 h at 37 °C with Alexa Fluor® 568 goat anti-rabbit IgG (Invitrogen A11036) 1:200 and Alexa Fluor® 488 goat anti-mouse IgG (Invitrogen A-11001) 1:100. Nuclei were stained with 4,6-Diamidino-2-Phenylindole (DAPI) Vectashield Mounting (Vector H-1200) and samples were observed through a confocal microscope Fluoview Olympus FV300. Software 4.3.

### Western blot

Nuclear acid proteins were separated on 18 % polyacrylamide SDS-PAGE gel while nuclear extract were separated on 10 % polyacrylamide SDS-PAGE gel and transferred to a nitrocellulose membrane according to the protocol described by Towbin et al., 1979 [12]. The membrane was exposed to Ponceau S to verify the efficiency of the transfer. The membrane was blocked with 5 % milk in PBS buffer- Tween-20 0.05 % (PBS-T) for 2 h at room temperature and then incubated with the anti-histone H3 antibody (Abcam ab1791) 1:5000, anti-histone H4 antibody (Santa Cruz sc-8658-R) 1:2000, anti-acetyl-histone H4 (Millipore 06-866) 1:4000, anti-histone H4 acetyl K12 antibody (Abcam ab61238) 1:2000, anti-histone H4 mono methyl R3 antibody (Abcam ab17339) 1:500, anti-trimethyl-histone H4 (Lys20) antibody (Millipore 07-463) 1:500, anti-lamin B1 antibody (Abcam ab16048) 1:5,000, anti-fibrillarin 1:3,000, anti-PRP6 1:500, all diluted with 2 % milk PBS-T overnight at 4 °C. The membranes were rinsed 3 times with PBS-T and incubated for 2 h with a horseradish peroxidase (HRP)-conjugated goat anti-rabbit IgG antibody (Thermo Fisher Scientific G-21234) 1:7,500 or goat anti-mouse IgG antibody (Thermo Fisher Scientific G-21040) 1:7,500 diluted in 2 % milk PBS-T. The antibody staining reaction on the membranes were developed by SuperSignal™ West Femto Maximum Sensitivity Substrate (Thermo Scientific 34095).

## Results

**Fig. 1:**
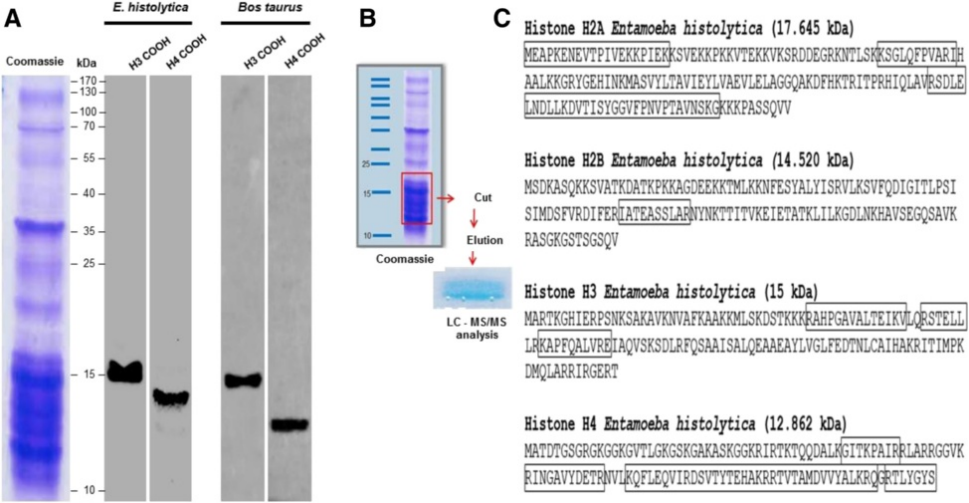
The histones H2A, H2B, H3 and H4 are present in the basic nuclear proteins from *E. histolytica.* **a** Basic nuclear extracts from *E. histolytica* were incubated with anti-H3 and anti-H4 antibodies. Histones from *Bos taurus* were used as a positive control and incubated with the same antibodies. **b** Flowchart representation of the approaches used to isolate the protein present between 10 and 20 kDa using gel electrophoresis. The recovered proteins were subjected to mass spectrometry. More than 100 proteins were identified by mass spectrometry. **c** Four proteins showed similarity to histones H2A, H2B, H3 and H4. The rectangle indicates the peptide identified for each histone

In eukaryotes, the NH_2_-terminus of histone H4 can undergo various post-translational modifications (PTMs), such as the acetylation of lysine residues 5, 8, 12 and 16, which are associated with transcriptional activation, and the trimethylation of lysine 20 of histone H4 (H4K20me3), which is associated with transcriptional repression [13]. To determine whether the amino-terminus of *Entamoeba* histone H4 also exhibits these PTMs, we initially obtained a crude preparation of histones. For this purpose, the nuclei of trophozoites were obtained, and an acid extraction was performed to obtain basic proteins, including histones. This preparation was separated using 18 % SDS-PAGE, and an enrichment of the fraction from 10 to 20 kDa was observed (Fig. 1a), suggesting that histones of *E. histolytica* were most likely present in this region. To confirm this assumption, WB assays were performed using commercial antibodies recognizing the COOH-terminus of histones H3 and H4. In the case of histone H3, a signal of approximately 15 kDa was observed, and in the case of histone H4, the antibody identified a protein of approximately 13 kDa. As a positive control for both antibodies, they were incubated with *Bos taurus* thymus histones. As expected, the antibodies recognized a 15 kDa band for histone H3 and band of 11.33 kDa for histone H4. These data indicate that the histones of this parasite are indeed present in the obtained preparation of basic nuclear proteins. To confirm that this preparation contained not only *E. histolytica* histones H3 and H4 but also histones H2A and H2B, we proceeded to recover proteins in the range of 10 to 17 kDa from the SDS-PAGE gel, which were then analyzed via mass spectrometry (Fig. 1b). The results revealed that the sample did include histones H2A, H2B, H3 and H4 (Fig. 1c). All of these data indicated that this basic nuclear preparation contained the four canonical histones [14]; none of the histone variants previously identified in other eukaryotes and protozoans were observed in this analysis [14, 15].

**Fig. 2:**
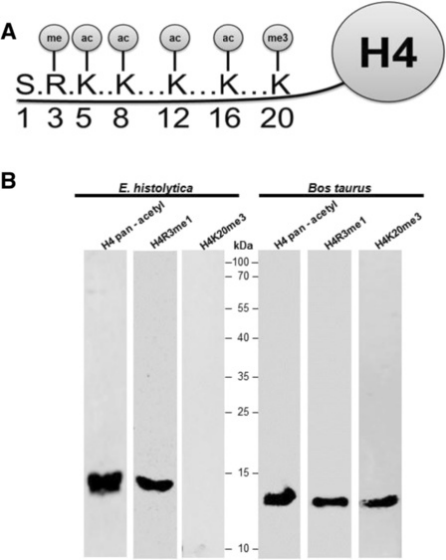
The N-terminal region of histone H4 from *E. histolytica* is acetylated in lysines 5, 8, 12 and 16 and mono-methylated in arginine 3. **a** Schematic representation of more common PTMs that occur at the N-terminal region of histone H4 in eukaryotic cells. **b** The basic nuclear proteins from *E. histolytica* were incubated with different antibodies. As a positive control, a histone preparation of *Bos taurus* was also incubated with the same antibodies

As stated previously, the amino-terminus of histone H4 exhibits three insertions compared with the primary structure of eukaryotic histone H4 (Additional file 1: Figure S1A). However, when these inserts are removed, the amino-terminus of *E. histolytica* shows a high homology (~81 %) with the amino-terminus of yeast and human histone H4 (Additional file 1: Figure S1B). Taking this into account, we decided to identify post-translational modifications occurring on histone H4 of this parasite using commercial antibodies. Alignment (Additional file 1: Figure S1C) of the peptides used to obtain a pan-acetyl antibody for *Tetrahymena thermophilus* with the N-terminal region of histone H4 from *E. histolytica* and *Homo sapiens* showed that lysines 5, 8, 12 and 16 are conserved at the amino-terminus of histone H4 of *E. histolytica* (Additional file 1: Figure S1C)*.* Thus, we decided to use this antibody to perform WB assays. The basic nuclear preparation was incubated with the pan-acetyl antibody, and a 13 kDa band was identified (Fig. 2b). *Bos taurus* histones were used as a positive control for the pan-acetyl histone antibody, which recognized an 11 kDa band (Fig. 2b). These results suggest that *E. histolytica* histone H4 is acetylated on one or more of the lysines located at positions 5, 8, 12 and 16. Another modification that occurs at the amino-terminus of histone H4 is the monomethylation of arginine 3 (H4R3me1) (Fig. 2a). Thus, the basic nuclear proteins obtained from *E. histolytica* were incubated with a polyclonal antibody against this epigenetic marker. As shown in Fig. 2b, this antibody again recognized a 13 kDa protein, corresponding to the previously established molecular weight of histone H4 and indicating that this marker exists on arginine 3 of this parasite, as observed in eukaryotes. In addition, this antibody also identified H4R3me1 among the histones of *Bos taurus*, with a weight of 11 kDa, as expected. In mammalian cells, the majority of histone H4 methylation is detected in the N-terminal tail on lysine 20 (H4K20) (Fig. 2a). This methylation mark is evolutionarily conserved from yeast to human and exists in three distinct states as mono-, di- and trimethylation. Each of these states results in distinct biological outputs: Mono- (H4K20me1) and dimethylated H4K20 (H4K20me2) are involved in DNA replication and DNA damage repair, whereas trimethylated H4K20 (H4K20me3) is a hallmark of silenced heterochromatic regions [16]. Because we are interested in identifying one heterochromatin marker in *E. histolytica* we proceeded to determine the presence of this PTM in the histone H4 of this parasite. Unexpectedly, no signal was found in extracts from *E. histolytica* (Fig. 2b), but there was a signal in the histones from *Bos taurus*. This result suggests that the H4K20me3 epigenetic repressive mark is likely not present in this parasite [17], because this epigenetic mark is enriched in telomeric region in eukaryotic cells and up to now the telomere in *E. histolytica* has not been identified. In conclusion, taking into account all of these data, we propose that the amino-terminus of histone H4 of *E. histolytica* harbors epigenetic marks associated with transcriptional activity, whereas no post-translational modification previously associated with transcriptional repression was identified.

### In the *E. histolytica* nucleus the activation and transcriptional repression marks converge

**Fig. 3:**
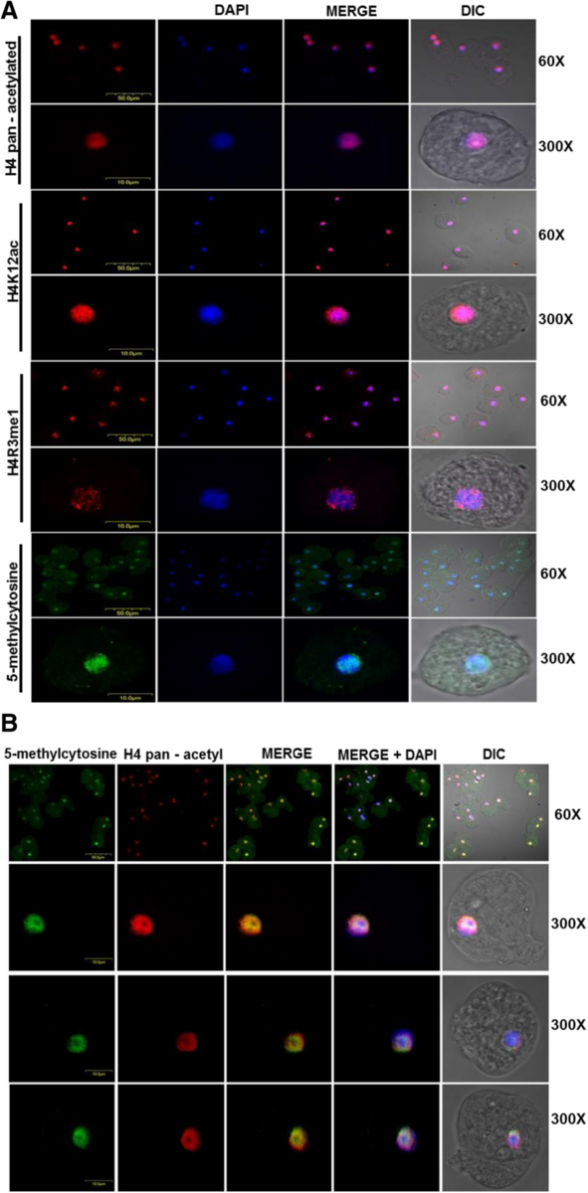
Nuclear localization of activation and repressive epigenetic marks in *E. histolytica* trophozoites. **a** Immunofluorescence assays with histone modification antibodies. IFAs were carried out with antibodies against specific histone modifications associated with transcriptional activation (H4 pan-acetylated, anti- H4K12ac, H4R3me1) and transcriptional repression (5-methyl-cytosine). Nuclei were stained with DAPI. The bar scale is indicated in each image. **b** Double IFAs using anti-mouse 5-methyl-cytosine (*green*) and pan-acetylated anti-rabbit H4 (*red*). 5-Methyl-cytosine signals were distributed in the nucleus and found to co-localize with anti-H4 pan-acetylated histone (*yellow*). The scale bar is given in each figure. A representative image from three independent experiments is shown

To determine if the previously identified lysine acetylation or mono-methylation of arginine 3 in histone H4 were located in different regions within the nucleus, immunofluorescence assays were performed using the pan-acetyl histone H4 antibody. The results show that this signal is present in all trophozoites, and when merged with DAPI, we found that this signal was located in the nucleus of all trophozoites (Fig. 3a). The amplification (300×) of one trophozoite image revealed that the acetylated lysines of histone H4 were distributed throughout the nucleus (Fig. 3a). Subsequently, an immunofluorescence assay was performed to determine whether each of the lysines identified by the pan-acetyl antibody (K5, K8, K12, and K16) was located in a specific region of the nucleus, such that the observed signals corresponded to the addition of each of the acetylated lysines. For this immunofluorescence assay, an antibody that specifically recognizes K12 of histone H4 was used. We again found that the signal for the acetylated K12 of histone H4 is present in all trophozoites. However, similar to the results obtained with the pan-acetyl H4 antibody, it was located throughout the nucleus (Fig. 3a). To assess whether the other epigenetic mark identified through WB (the mono-methylation of arginine 3 of histone H4, which has been shown to be associated with transcriptional activation) was also distributed throughout the nucleus or was included among the acetylated lysines, IFA was performed (Fig. 3a). Once again, the signal obtained with this antibody was present in all nuclei, showing a distribution throughout the nucleus when observed at a higher resolution (300×) (Fig. 3a). All of these data suggest that activation marks (such as acetylation and/or methylation) present in this parasite are distributed throughout the *E. histolytica* nucleus.

Because our WB assays with the anti-H4K20me3 antibody resulted in no signal, we were not able to use this antibody to identify heterochromatin regions in this parasite [17]. For this reason and considering that histone methylation and DNA methylation are mechanisms that act in concert and that the presence of DNA methylation has previously been demonstrated in this parasite [18], we decided to use an antibody that recognizes methylated cytosines (anti-5-methyl cytosine) to indirectly locate transcriptionally inactive regions in amoebas (Fig. 3a). Our data indicated that this mark was present in most trophozoites, and merging with DAPI showed that it was also present in the nucleus. Unexpectedly, the amplification of some of these signals (300×) from three independent experiments showed that the methylated DNA was distributed throughout the nucleus (Fig. 3a). In order to establish if the euchromatin marks (pan-acetyl histone H4) and DNA methylation marks overlapped, IFAs were performed with anti-pan-acetyl histone H4 and anti-5-methyl-cytosine histone H4 antibodies. The results of the immunofluorescence analyses indicated that the activation and transcriptional repression marks co-localize in *E. histolytica* (Fig. 3b).

### The *E. histolytica* nucleus contains at least two types of nuclear bodies

Studies aimed at determining how the nucleus is physically and functionally organized have revealed that it is very organized and highly dynamic. A prominent feature of the nuclear landscape is its ability to harbor a variety of discrete subnuclear organelles, collectively referred to as nuclear bodies. Nuclear bodies spatially compartmentalize the nuclear environment and create different sites where proteins and RNAs concentrate, which streamlines biological processes such as replication, DNA repair and messenger RNA maturation [19].

**Fig. 4:**
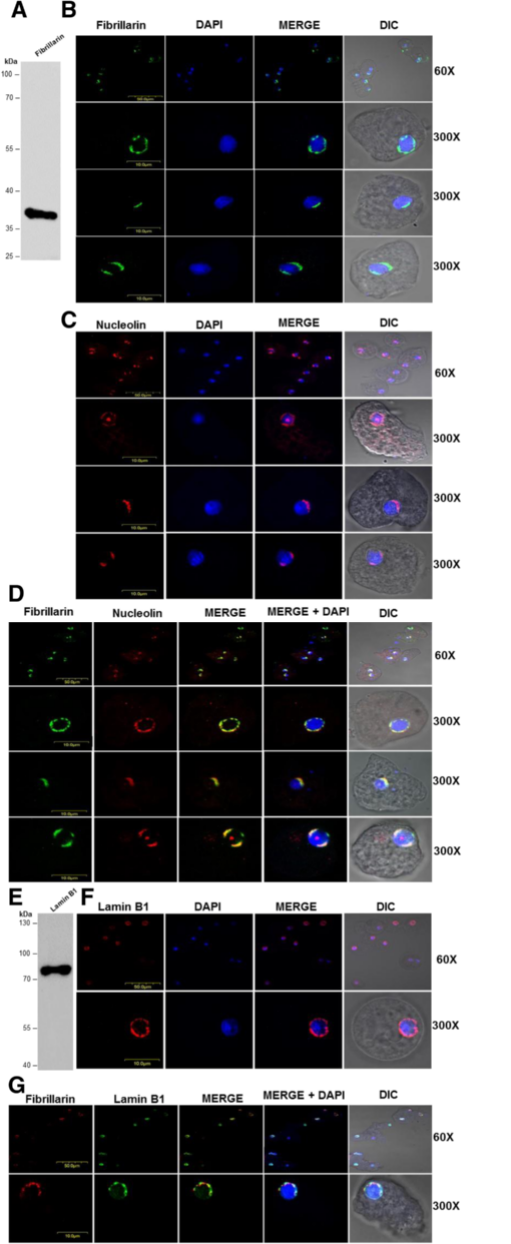
The nucleolus is a nuclear body located at the nuclear periphery of *E. histolytica.* **a** Anti-fibrillarin antibody was incubated with nuclear extract from *E. histolytica.* A protein of approximately 35 kDa was identified. **b** Indirect Immunofluorescence assays (IFAs) were performed using anti-fibrillarin antibody (*green*), and the nuclei were stained with DAPI (*blue*). Three different signal patterns were obtained with this antibody. In the first, the nucleus was found to be located in the periphery of the nucleus. In the second, the fibrillarin was located to one side of the nucleus, while in the third, the fibrillarin was found in two poles of the nucleus. Representative IFAs of the three types of signals are shown. **c** IFA analysis of nucleolin. The nucleolin antibody displays three different patterns: in the majority of trophozoites (50 %), the nucleolin is present at the nuclear periphery. In other images (35 %), the nucleolin is present at one pole of the nucleus. Finally, in the remaining trophozoites, the nucleolin is located on 2 sides of the nucleus (15 %). The nucleus was detected by DAPI staining (*blue*). The scale bar is indicated in each image. **d** Dual-color IFA with anti-mouse fibrillarin (*green*) and anti-rabbit nucleolin (*red*). Fibrillarin and nucleolin co-localize in the nucleus of *E. histolytica* in the three types of signals (*yellow*). **e** Western blot assay. Anti-lamin B1 antibody was incubated with nuclear extracts from *E. histolytica* and recognized a protein of approximately 78 kDa. **f** IFAs were carried out with antibodies against lamin B1. The nuclei were stained with DAPI (*blue*). The IFA showed that lamin is located around the nucleus in *E. histolytica*. **g** Fibrillarin (*red*) and anti-lamin B1 (*green*) co-localize (*yellow*) at the nuclear periphery in *E. histolytica* only when the fibrillarin is present around the nucleus. The scale bar is indicated in each figure. A representative image of three independent experiments is shown

To determine whether there are also nuclear bodies in the *E. histolytica* nucleus, we employed antibodies against two proteins specific to the nucleolus: fibrillarin (which was kindly provided by Dr. Miguel Ángel Vargas) and nucleolin. Initially, nuclear *E. histolytica* trophozoite extracts were assessed via WB to validate the anti-fibrillarin antibody. A protein of the expected size of approximately 35 kDa was identified (Fig. 4a). Based on these results, both antibodies were used to perform IFAs. Figure 4b and c show that the anti-nucleolin and anti-fibrillarin antibodies produced three types of signals. One of the signal patterns, identified in 50 of 100 parasite cells, showed fibrillarin and nucleolin to be largely located at the periphery of the nucleus. In 35 of the cells, the two proteins were instead located at both poles of the nucleus, and in 15 of the cells, the proteins were located at just one end of the nucleus (Fig. 4b and c). For nucleolin, in addition to producing three types of signals, as was observed for fibrillarin (Fig. 4c), other signals could also be identified because this protein is not only a constituent of the nucleolus in eukaryotic organisms but also performs other functions in the nucleus [20]. Therefore, IFAs were performed to determine if nucleolin and fibrillarin co-localize. The overlap of the two proteins corroborated the existence of a nucleolus not only at the periphery of the nucleus of *Entamoeba* (Fig. 4d), but also in one or two poles of the nucleus, two patterns not described previously (Fig. 4d) [21]. Finally, a mouse anti-lamin B1 antibody was used to demonstrate that the nucleolus is located at the nuclear periphery. Initially, we decided to determine whether the mouse anti-lamin B1 antibody recognized a lamin-like protein in *E. histolytica* nuclear extracts via WB assays (Fig. 4e). The mouse anti-lamin B1 antibody recognized a protein of approximately 78 kDa in the nuclear extracts of *E. histolytica* (Fig. 4e). Thus, we proceeded to use this antibody in further IFAs: the signal obtained with the anti-lamin B1 antibody was located across the entire surface of the nucleus, and when superimposed on the image of nuclei stained with DAPI, we could clearly distinguish the nucleus from the cytoplasm (Fig. 4f). More importantly, the signal suggested the existence of a lamin-B-like protein in the amoeba. Finally, a co-localization assay was performed with anti-lamin B1 and anti-fibrillarin, which revealed that the two signals co-localized at the periphery of the *E. histolytica* trophozoite nucleus (Fig. 4g). This finding confirmed that the nucleolus shows the perinuclear localization in the amoeba.

### The *E. histolytica* nucleus contains at least two types of nuclear bodies

**Fig. 5:**
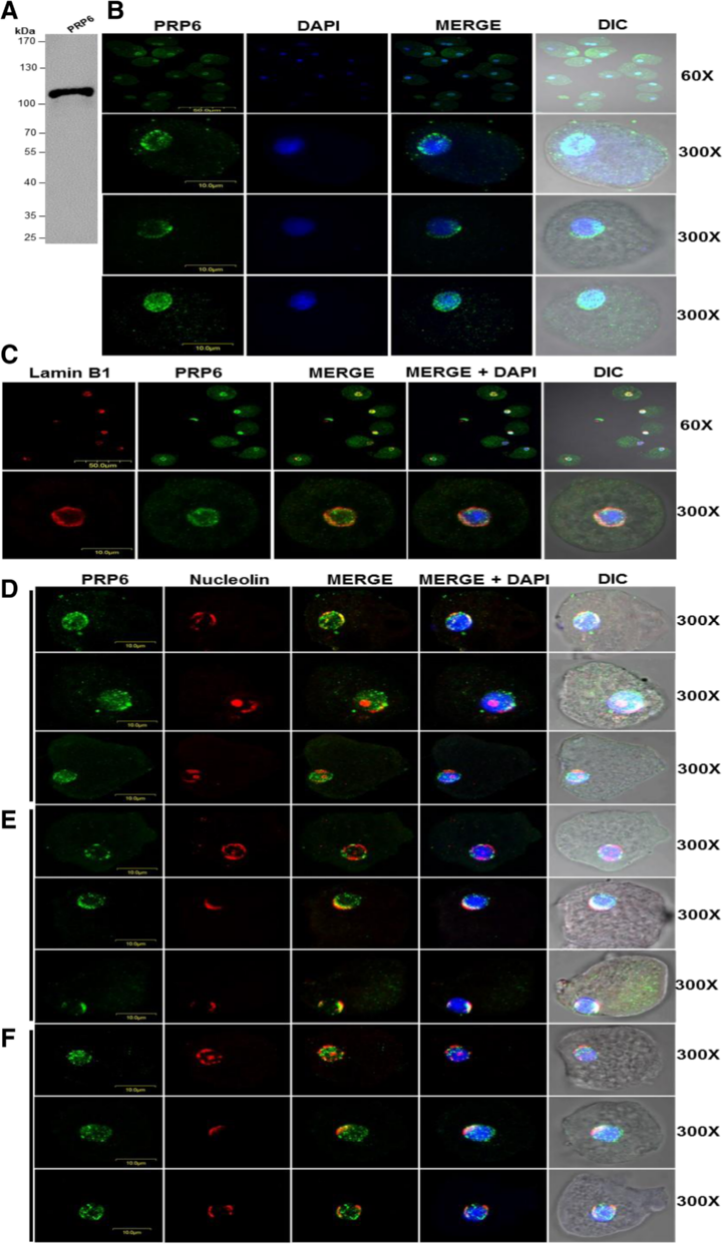
Nucleolus and speckles are present in the nucleus of *E. histolytica.* **a** Western blot analysis of nuclear extracts from *E. histolytica* with anti-PRP6 antibody. **b** IFA analysis of PRP6 (*green*) is observed as a punctuated pattern. The nucleus was detected by DAPI (*blue*) staining. Anti-PRP6 antibodies give three different signals. In the first, PRP6 is located in the nuclear periphery and also in the nucleus (40 % of trophozoites). The second signal is found in the nuclear periphery (40 %), and the last signal is distributed only into the nucleus (20 %). The scale bar is indicated in each image. **c** IFAs were carried out with antibodies against lamin B1 (*red*) and PRP6 (*green*). The nuclei were stained with DAPI (*blue*). Co-localization signals (*yellow*) indicate that the PRP6 signal is located inside and around the nucleus (300×). **d** Dual color IFA with anti-mouse PRP6 (*green*) and anti-rabbit nucleolin (*red*). The PRP6 signal (*green*) is present around and inside the nucleus. The nucleolin signal is found at the nuclear periphery but also in one or both poles of the nucleus. The PRP6 and nucleolin signals co-localized only when PRP6 is distributed around the nucleus (*yellow*). **e** The PRP6 signal (*green*) is located mainly at the nuclear periphery and co-localized with nucleolin (*red*) when it is distributed around the nucleus or in one or both poles of the nucleus (*yellow*). **f** The PRP6 signal (*green*) is found inside the nucleus and is not co-localized with the nucleolin (*red*) signal when it is situated at the nuclear periphery in one or both poles of the nucleus

To identify other common types of non-membranous nuclear bodies that are present in many eukaryotic organisms, such as speckles which are nuclear domains enriched in pre-mRNA splicing factors, we decided to use RNA processing proteins (PRPs), a type of organelle-specific protein. The *E. histolytica* PRP6 protein was described in 2000 by the group of Dr. Vargas Mejia, and we employed the antibody obtained in that study to perform WB assays and verify its functionality [22]. The anti-PRP6 antibody recognized a protein of the expected size of approximately 105 kDa (Fig. 5a), as previously described [22]. Thus, this antibody was used in further immunofluorescence assays. The signal corresponding to anti-PRP6 (speckled structures) was present in all trophozoite nuclei. However, amplification of some of the obtained images (300×) showed that this protein presented three types of signals. One signal type occurred both within the nucleus and at the periphery (40 %), the second signal type was observed only at the periphery (40 %), and the third signal type was only present within the nucleus (20 %), (Fig. 5b). To demonstrate that the signal was effectively both inside and at the periphery of the nucleus, but not in the cytoplasm, another immunofluorescence assay was performed using the anti-PRP6 and anti-lamin B1 antibodies. The result indicated that the PRP6 signal was both within the nucleus and at its periphery (Fig. 5c).

Lastly, to establish whether the nucleolus and speckles occupied different locations within the nucleus, IFAs using the anti-PRP6 and anti-nucleolin antibodies were performed. Figure 5d shows that PRP6 and nucleolin occupied a different site within the nucleus only when PRP6 was located inside the nucleus (Fig. 5f). However, when PRP6 was located in the nuclear periphery and also in the nucleus (Fig. 5d) or only in the nuclear periphery Fig. 5e), it co-localized with nucleolin. Taking these data together, we can suggest that there are at least two dynamic nuclear bodies in the *E. histolytica* nucleus: the nucleolus and speckles.

## Discussion

To complete its life cycle, *E. histolytica* must differentiate into a mobile and invasive form, the trophozoite, and an infectious form, the cyst. During its differentiation process, the parasite must regulate genes in a phase-specific manner to allow it to complete its life cycle. However, the molecular mechanisms that regulate *E. histolytica* gene expression have been poorly studied. Transcription has been shown to represent a significant control point in higher eukaryotes, where chromatin is involved in the regulation of gene expression [23]. Experimental evidence indicates that chromatin may also regulate gene expression in *E. histolytica*, as it has been demonstrated that *E. histolytica* DNA is organized into chromatin and that the nucleosome (the fundamental unit of chromatin) consists of four canonical histone dimers. In addition, *E. histolytica* produces proteins that acetylate, deacetylate and methylate histones [6, 8, 24], which are responsible for chromatin structure modifications. Post-translational modifications of histones and DNA methylation are two of the epigenetic mechanisms that have been best studied in eukaryotic organisms. Both mechanisms have already been described in *E. histolytica*. However, the residues of histones H3 or H4 upon which these post-translational modifications occur as well as their localization in the nucleus of this parasite are unknown. To answer these questions, a crude preparation of histones from this parasite was obtained, but despite using protocols and resins previously employed in other eukaryotes and other parasites, a pure preparation of histones that would allow us to perform mass spectrometry assays and to identify post-translational modifications of amoeba histones could not be recovered. We believe that the differences in the isoelectric point of the *Entamoeba* histones (as they are less basic, according to the Compute Pi/Mw tool program) compared with those of humans could have interfered with the purification of the *E. histolytica* histones. Another unexpected result was that only peptides corresponding to the 4 canonical histones were identified through the mass spectrometry analysis of the crude preparation of *E. histolytica* histones, and no histone variants were identified. In addition, an *in silico* analysis of the data bank for this parasite did not return any results. Thus, it is necessary to establish an improved methodology for purifying the histones of this parasite, which would allow not only the identification of post-translational modifications occurring at the terminal ends of *Entamoeba* histones but also the enrichment of histone variants from this parasite (if any exist). If no such variants are found, it would indicate that *E. histolytica* only exhibits canonical histones. This contrasts with previous reports on other parasites, such as *Plasmodium falciparum* and *Trypanosoma brucei*, in which histone variants have been identified (e.g., H.3.3, CenH3, H2AZ and histone H2AX) and their role in regulating gene expression subsequently demonstrated [15, 25–28].

Interestingly, in this study, we found that when the insertions present at the amino-terminus of histone H4 of *E. histolytica* were removed, this histone was virtually identical (81 %) to that of *Saccharomyces cerevisiae*. Considering that this histone exhibits lysine residues at positions 5, 8, 12 and 16 that can be acetylated [13], we decided to use a pan-acetyl antibody. This demonstrated for the first time that the amino-terminus of histone H4 is acetylated at lysines 5, 8, 12 and 16 and monomethylated at arginine 3. In contrast, commercial antibodies recognizing tri-methylated lysine 20 failed to identify this mark. However, the absence of this PTM could be due to the enrichment of H4K20me3 in telomeric heterochromatin [29, 30]. The fact that *E. histolytica* chromosomes are circular and linear and that telomeric sequences have not been identified in the linear chromosomes could explain the absence of this epigenetic mark.

In this study, the nuclear localization of the identified epigenetic marks was also determined. The obtained data on the *E. histolytica* marks suggest that the activation and repression signals converge. This finding is in contrast to previous descriptions in many organisms, from yeast to humans, as well as in two of the best-studied parasites, *P. falciparum* and *T. brucei*, and suggests that there is no compartmentalization in the nucleus of *E. histolytica*. This finding is very interesting because in the case of *P. falciparum*, it has been reported that the nucleus is compartmentalized into a central region rich in epigenetic marks associated with transcriptional activation (H3K4me3 and pan-acetyl histone H4), a perinuclear repression center region enriched in marks of repression such as H3K9me3 and histone deacetylase *PfSir2*, and the site of expression of *var* genes and the nucleolus [31, 32]. A similar situation has been established in *T. brucei*, which also exhibits a compartmentalized nucleus [33]. This compartmentalization is involved in regulating the expression of genes involved in antigenic variation, as observed in *P. falciparum*; these genes are VSG for *T. brucei* and PfEMP-1 for *P. falciparum*. Thus, if these data are corroborated, the nuclear architecture may not function as another epigenetic mechanism that regulates gene expression in *E. histolytica*. This could be because *E. histolytica* diverged very early and nuclear compartmentalization may have arisen later as parasite life cycles became more complex, requiring more finely regulated expression of their genes in a host, organ-specific manner.

Another interesting finding involved the identification of a possible lamin B1 in *E. histolytica*. Lamin plays important roles in many nuclear processes, including transcription, DNA replication, cell cycle control and DNA repair [23]. For quite some time, it was believed that lamin was an exclusive metazoan compartment. However, a protein similar to lamin, designated Nup-1, was recently identified in *T. brucei* [34], *E. invadens*, *Gregarina melanopli*, *Euglena gracilis*, *Giardia* and *Trichomonas*, indicating that lamin is not exclusive to metazoans [35]. Thus, the 78 kDa protein identified in *E. histolytica* in this study that might be lamin-like may also be involved in regulating gene expression by interacting with chromatin, as reported in eukaryotes and *T. brucei*. Therefore, the development of parasites in which this protein is knocked down will be necessary to allow us to elucidate their participation in genome organization and, thus, in regulating gene expression in *E. histolytica*. Additionally, techniques such as chromatin conformation capture (3C) must be implemented to map the contact between chromatin and this lamin B-like protein.

In this study, two functionally distinct types of nuclear bodies were also visualized: the nucleolus and speckles. The presence of a nucleolus was previously demonstrated at the nuclear periphery in *E. histolytica* [21]. In the present study, two signals, plus one signal located at one end of the nucleus and another located at both ends of the nucleus, were identified. Cell cycle-dependent dynamic organization of the nucleolus has been demonstrated in *P. falciparum*, in which the rDNA is located at one end of the nucleus during the ring stage. However, during the replication stages, the rDNA disintegrates into individual units and is observed as multiple foci in the nucleolus. This demonstrates that rDNA clustering is cell cycle-dependent [36]. Thus, we propose that the nucleolus of *E. histolytica* is also a highly dynamic cell- and cycle-dependent type of nuclear body. Speckles are another highly conserved type of nuclear body. In eukaryotic organisms, speckles are subnuclear structures that act as compartments that can provide splicing factors to active transcription sites, which are observed in the nucleus as irregular dotted structures that vary in size and shape [37]. Considering that speckles are highly conserved nuclear bodies, we decided to determine whether speckles might be present in the nucleus of *Entamoeba*, as it was previously found that *E. histolytica* has 3000 introns among 9938 identified genes [38]. In addition, some of the factors that constitute the spliceosome of this parasite have been recently reported [39]. These results suggest the existence of this nuclear body, which could provide the splicing factors needed to remove introns from *E. histolytica* mRNA. We are currently attempting to demonstrate the relationship between this nuclear body and the RNA pol II transcription machinery using 5-bromouridine 5-triphosphate (BrUTP) incorporation assays. Finally co-localization assays between nucleolin and PRP6 indicate that when PRP6 is inside the nucleus, it is not co-localized with nucleolin (nucleolus). However, when the PRP6 signal is located in the nuclear periphery or in a pole of the nucleus, both proteins co-localize. These results suggest that while the nucleolus and speckles identified through these proteins are two nuclear bodies, the proteins that constituted both nuclear bodies are highly dynamic and may follow specific pathways between different nuclear bodies prior to activation. Indeed, recent experimental evidence suggests that specific proteins of an organelle may not only reside in the nuclear body in which they work but also travel between different nuclear bodies [40, 41].

## Conclusions

In this work, we demonstrated for first time that lysines 5, 8, 12 and 16 of *E. histolytica* histone H4 are acetylated and that arginine 3 is monomethylated. The location of these marks in the nucleus of this parasite suggest (but are not conclusive), that unlike other protozoans and eukaryotes, the activation and repression marks co-localized. Finally, the presence of at least two types of nuclear bodies, nucleolus and speckles, was demonstrated. Thus, this study provides new tools that can be used in various tests, such as chromatin-immunoprecipitation (ChIP), to determine which genes are regulated by pan-acetyl histone H4 and to study the role of lamin in processes such as replication, cell division and the organization of chromatin via 3C assays. The challenge is now to understand how these epigenetic marks affect chromatin and how the nuclear bodies work together to regulate gene expression in this parasite.

## Additional file

Additional file 1: Figure S1. The N-terminal region of histone H4 of E. histolytica is conserved among different eukaryotic cells. A) Alignment of the N-terminal region of histone H4 from *E. histolytica* (*Eh*), *P. falciparum* (*Pf)*, *Saccharomyces cerevisiae* (*Sc)*, *Drosophila melanogaster* (*Dm*), and *Homo sapiens* (*Hs*). (*). Identical residues; (:) compensatory changes. Three insertions present in the N-terminal region of histone H4 of *E. histolytica* are indicated in red letters. B) Elimination of the three insertions present in the N-terminal region of histone H4 of *Eh* (indicated by lines) generates a highly conserved N-terminal region among *Eh*, *Pf*, *Sc*, *Dm* and *Hs*. Alignment of the H4 N-terminal region of *Eh*, *Pf*, *Sc*, *Dm* and *Hs* with H4 pan-acetylated peptide from *Tetrahymena thermophiles* and a H4 arginine 3 mono-methylated peptide are shown. Rectangle indicated the most conserved residues identified for both peptides. Lysines and arginine amino acids susceptible to be acetylated are indicated in bold. (DOCX 27 kb)

## Abbreviations

ChIP: Chromatin-Immunoprecipitation
BrUTP: 5-bromouridine 5-triphosphate
H3K4me3: Histone 3 lysine 4 trimethylation
H3K27me2: Histone 3 lysine 27 dimethylation
H4R3me2: Histone 4 arginine 3 dimethylation
H4K20me3: Histone 4 lysine 20 trimethylation
HATs: histone acetyltransferases
HKMTs: lysine methyl transferases
PRMTs: arginine methyl transferases
