# Supplementary material for: Identification of repressive and active epigenetic marks and nuclear bodies in Entamoeba histolytica
Source: Parasit Vectors. 2016 Jan 14;9:19. doi: 10.1186/s13071-016-1298-7 (PMC4712492; doi:10.1186/s13071-016-1298-7)
Supplement: Additional file 1: Figure S1. — The N-terminal region of histone H4 of E. histolytica is conserved among different eukaryotic cells. A) Alignment of the N-terminal region of histone H4 from E. histolytica (Eh), P. falciparum (Pf), Saccharomyces cerevisiae (Sc), Drosophila melanogaster (Dm), and Homo sapiens (Hs). (*). Identical residues; (:) compensatory changes. Three insertions present in the N-terminal region of histone H4 of E. histolytica are indicated in red letters. B) Elimination of the three insertions present in the N-terminal region of histone H4 of Eh (indicated by lines) generates a highly conserved N-terminal region among Eh, Pf, Sc, Dm and Hs. Alignment of the H4 N-terminal region of Eh, Pf, Sc, Dm and Hs with H4 pan-acetylated peptide from Tetrahymena thermophiles and a H4 arginine 3 mono-methylated peptide are shown. Rectangle indicated the most conserved residues identified for both peptides. Lysines and arginine amino acids susceptible to be acetylated are indicated in bold. (DOCX 27 kb) [file 13071_2016_1298_MOESM1_ESM.docx]

10 20 30 40 50 60 70 80 90 100 110

**A**

**B**

H4Eh MATDTGSGRGKGGKGVTLGKGSKGAKASKGGKRIRTKTQQDALKGITKPAIRRLARRGGVKRINGAVYDETRNVLKQFLEQVIRDSVTYTEHAKRRTVTAMDVVYALKRQGRTLYGYS-

H4Pf -----MSGRGKGGKG--LGKG--------GAKRHR-KILRDNIQGITKPAIRRLARRGGVKRISGLIYEEIRGVLKVFLENVIKDSIMYTEHAKRKTVTAMDIVYSLKRQGRTLYGFGG

H4Sc -----MSGRGKGGKG--LGKG--------GAKRHR-KILRDNIQGITKPAIRRLARRGGVKRISGLIYEEVRAVLKSFLESVIRDSVTYTEHAKRKTVTSLDVVYALKRQGRTLYGFGG

H4Dm -----MTGRGKGGKG--LGKG--------GAKRHR-KVLRDNIQGITKPAIRRLARRGGVKRISGLIYEETRGVLKVFLENVIRDAVTYTEHAKRKTVTALDVVYALKRQGRTLYGFGG

H4Mm -----MSGRGKGGKG--LGKG--------GAKRHR-KVLRDNIQGITKPAIRRLARRGGVKRISGLIYEETRGVLKVFLENVIRDAVTYTEHAKRKTVTAMDVVYALKRQGRTLYGFGG

H4Hs -----MSGRGKGGKG--LGKG--------GAKRHR-KVLRDNIQGITKPAIRRLARRGGVKRISGLIYEETRGVLKVFLENVIRDAVTYTEHAKRKTVTAMDVVYALKRQGRTLYGFGG

:******** **** *.** * * :* ::**************** ***.* :*:* * *** ***.**:*:: *******:***::*:**:**********:.

**B**

**B**

10 20 30 40 50 60 70 80 90 100 110

H4Eh MATDTGSGRGKGGKGVTLGKGSKGAKASKGGKRIRTKTQQDALKGITKPAIRRLARRGGVKRINGAVYDETRNVLKQFLEQVIRDSVTYTEHAKRRTVTAMDVVYALKRQGRTLYGYS-

H4Eh MSG**R**G**K**GG**K**GLG**K**GGG**K**RIRTKTQQDALKGITKPAIRRLARRGGVKRINGAVYDETRNVLKQFLEQVIRDSVTYTEHAKRRTVTAMDVVYALKRQGRTLYGYS-

H4Pf MSGRGKGGKGLGKGGAKRHR-KILRDNIQGITKPAIRRLARRGGVKRISGLIYEEIRGVLKVFLENVIKDSIMYTEHAKRKTVTAMDIVYSLKRQGRTLYGFGG

H4Sc MSGRGKGGKGLGKGGAKRHR-KILRDNIQGITKPAIRRLARRGGVKRISGLIYEEVRAVLKSFLESVIRDSVTYTEHAKRKTVTSLDVVYALKRQGRTLYGFGG

H4Dm MTGRGKGGKGLGKGGAKRHR-KVLRDNIQGITKPAIRRLARRGGVKRISGLIYEETRGVLKVFLENVIRDAVTYTEHAKRKTVTALDVVYALKRQGRTLYGFGG

H4Mm MSGRGKGGKGLGKGGAKRHR-KVLRDNIQGITKPAIRRLARRGGVKRISGLIYEETRGVLKVFLENVIRDAVTYTEHAKRKTVTAMDVVYALKRQGRTLYGFGG

H4Hs MSGRGKGGKGLGKGGAKRHR-KVLRDNIQGITKPAIRRLARRGGVKRISGLIYEETRGVLKVFLENVIRDAVTYTEHAKRKTVTAMDVVYALKRQGRTLYGFGG

H4Tt pan-acetylated peptide --AGG**K**GG**K**GMG**K**VGA**K**RHS------------------------------------------------------------------------------------

H4R3me1Hs peptide --SG**R**GKGGKGLGKGGAKRHR-KVLRDNIQGITKPAIRRLARRGGVKRISGLIYEETRGVLKVFLENVIRDAVTYTEHAKRKTVTAMDVVYALKRQGRTLYG---

.******:** *.**
